# Conjugates of Copper Alginate with Arginine-Glycine-Aspartic Acid (RGD) for Potential Use in Regenerative Medicine

**DOI:** 10.3390/ma13020337

**Published:** 2020-01-11

**Authors:** Justyna Fraczyk, Joanna Wasko, Malgorzata Walczak, Zbigniew J. Kaminski, Dorota Puchowicz, Irena Kaminska, Maciej Bogun, Marcin Kolasa, Ewa Stodolak-Zych, Anna Scislowska-Czarnecka, Beata Kolesinska

**Affiliations:** 1 Institute of Organic Chemistry, Faculty of Chemistry, Lodz University of Technology, Zeromskiego 116, 90‐924 Lodz, Poland; justyna.fraczyk@p.lodz.pl (J.F.); joanna.wasko@edu.p.lodz.pl (J.W.); malgorzata.walczak@p.lodz.pl (M.W.); zbigniew.kaminski@p.lodz.pl (Z.J.K.); 2 Lukasiewicz Research Network-Textile Research Institute, Brzezinska 5/15, 92-103 Lodz, Poland; puchowicz@iw.lodz.pl (D.P.); ikaminska@iw.lodz.pl (I.K.); mbogun@iw.lodz.pl (M.B.); 3 Military Institute of Hygiene and Epidemiology Department of Pharmacology and Toxicology, Kozielska 4, 01-163 Warsaw, Poland; marcinkolasa@wp.pl; 4 Department of Biomaterials, AGH‐University of Science and Technology, A. Mickiewicz 30, 30-059 Krakow, Poland; stodolak@agh.edu.pl; 5 Academy of Physical Education, Department of Physiotherapy, Section of Anatomy, 31-008 Krakow, Poland; anna.scislowska@awf.krakow.pl

**Keywords:** copper alginate, RGD derivatives, polysaccharide-peptide conjugates, antibacterial activity, cytotoxicity

## Abstract

Current restrictions on the use of antibiotics, associated with increases in bacterial resistance, require new solutions, including materials with antibacterial properties. In this study, copper alginate fibers obtained using the classic wet method were used to make nonwovens which were modified with arginine-glycine-aspartic acid (RGD) derivatives. Stable polysaccharide-peptide conjugates formed by coupling with 4-(4,6-dimethoxy-1,3,5-triazin-2-yl)-4-methylmorpholinium toluene-4-sulfonate (DMT/NMM/TosO^−^), and materials with physically embedded RGD derivatives, were obtained. The materials were found to be characterized by very high antibacterial activity against *S. aureus* and *K. pneumoniae*. Cytotoxicity studies confirmed that the materials are not cytotoxic. Copper alginate conjugates with RGD peptides have strong potential for use in regenerative medicine, due to their biocompatibility and innate antibacterial activity.

## 1. Introduction

**Figure 1:**
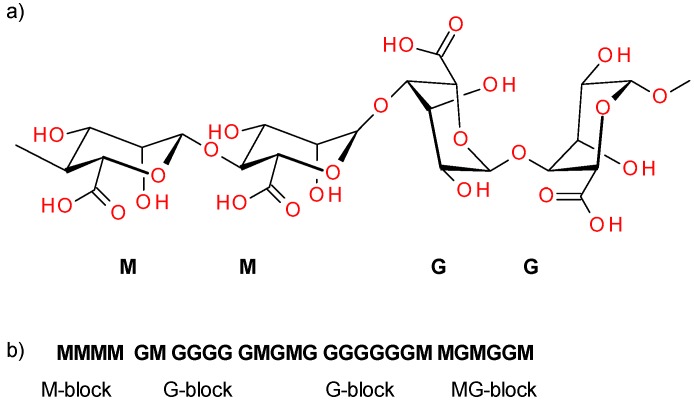
Structure of β-D-mannuronic acid-β-D-mannuronic acid-α-l guluronic acid-α-l guluronic acid (MMGG) fragment of alginic acid (**a**), distribution of M and G blocks in the polysaccharide chain (**b**).

Alginate is a naturally occurring polysaccharide composed of (1→4) linked β-D-mannuronic acid (M) and its C-5 epimer α-l guluronic acid (G). It consists of a mixture of GG, MG, and MM blocks, arranged in an irregular blockwise pattern that determines their properties [1] (Figure 1). Due to the abundance of algae in water reservoirs, they are a predominant form of biomass. Their content in biomass is estimated at up to 40% of dry weight. The global production of alginate is 50,000 tons per year. Almost 30% of this is used by the food industry, while the remainder is employed in industrial, pharmaceutical, biotechnological, and dental applications [2].

Alginic acid is insoluble in water but forms water-soluble salts with monovalent cations, while divalent metal salts (Ca^2+^, Sr^2+^ and Ba^2+^) of alginic acid are again insoluble in water. These properties are employed to isolate alginic acid from algae. The solubility of alginic acid in water depends on the pH of the solution, the ionic strength of the medium, and the presence of ions conditioning gelation in the solvent. As has been mentioned, alginates form acid gel and chelates with divalent cations that act as crosslinkers between adjacent polymer chains. The presence of G blocks is responsible for ionic interactions, while MG blocks are involved in the formation of weak bonds [3]. The affinity of alginate towards divalent cations is known to decrease in order as follows: Pb > Cu > Cd > Ba > Sr > Ca > Co > Ni,Zn > Mn. However, the Ca^2+^ ion is most often used to prepare alginate gel. Due to their unique physicochemical properties, alginic acids and their salts have found a wide range of applications in pharmaceuticals [4,5], medicine [6,7], agriculture [8], and purification processes [9,10,11]. Alginate has many hydroxyl and carboxyl groups located along the polymer backbone, so it can be modified easily, allowing the construction of new materials with improved or new structures and functional properties. The chemical modification of alginate can be carried out by various methods. Polycondensation reactions (esterification, amidation) are the most common, but other processes such as amination and grafting by radical polymerization are also used.

The most important requirement for the use of alginates as a biomaterial is their ability to provide a physically and chemically favorable environment for living cells [12]. Alginates have many advantages as biomaterials, including hydrophilicity, biocompatibility, and lack of immunogenicity [13]. The susceptibility of alginates to form gels capable of encapsulating cells, drugs, and other active biological compounds is another important property. However, an important disadvantage is that alginates are not cell adhesive [14]. For this reason, chemical functionalization with cell signaling compounds or adhesion enhancing compounds is crucial to overcome the low affinity of alginates towards cells. In addition to enhancing cellular interactions, functionalization can also play a role in controlling cell growth, differentiation, and behavior. The extensive use of RGD peptides to modulate the properties of alginate-based materials is due to the fact that these derivatives are by far the most effective peptide compound stimulating cell adhesion. The RGD sequence is a structural motif characteristic of many proteins in living organisms, which activates the adhesion of more than one type of cell. It does this by acting on more than one type of receptor, stimulating adhesion and improving cell survival. In multicellular organisms, cell–cell interactions and cell–extracellular matrix (ECM) interactions occur via adhesion receptors. In the group of cell adhesion receptors, the main role is played by various integrins. More than half of the 24 known integrins bind ECM proteins containing the RGD motif. Given that integrin-modulated adhesion affects the regulation of many physiological processes [15] (cell migration, proliferation, survival, apoptosis) as well as pathological processes (e.g., tumor invasion, metastasis), learning about the ligands that are selective and specific for these receptors is crucial to the rational design of materials for regenerative medicine [16,17,18,19,20]. Selective integrin ligands are used as inhibitors of tumor cell angiogenesis, when overexpression of integrins is observed [21,22], and as blockers of the blood coagulation process. Modified integrin ligands have also been used as radioisotope or dye transport systems in cancer diagnostics (positron-emission tomograph (PET), single photon emission computed tomography (SPECT), fluorophores) [23] and for the production of new biomaterials with increased cell adhesion [24,25].

Modification of alginate with polar bioactive compounds, including peptides, is traditionally performed using carbodiimides (1-ethyl-3-(3-dimethylaminopropyl)carbodiimide (EDC)/N-hydroxysuccinimide (NHS) is particularly widely used). The average coupling efficiency ranges between 0.1 and 1.0 mol% of peptide per uronate monomer [26]. Although this concentration of peptides induces the deposition and interaction of modified alginates with myoblasts [27], olfactory cells [28], mesenchymal stem cells [29], and endothelial cells [30], increasing the concentration of peptides affects both the efficiency of cell binding and cell differentiation, as demonstrated by Rowley and Mooney in their studies of C2C12 myoblasts and RGD modified alginate [27]. Similar observations have been made for alginate modified with YIGSR peptide and neuroblastoma cells [31]. The use of carbodiimides as condensing reagents also involves the formation of urea and N-acylurea derivatives of the alginate, which result from the activation of the carboxylic function of water-soluble natrum alginate. This contributes to the impairment of gelling properties and simultaneously to a lack of biological activity in the peptide. However, another important disadvantage of alginate is that, with a low degree of substitution (DS), coupling reactions with carbodiimides are more selective for G residues in comparison to M residues, while G residues adjacent to M residues are preferred to two adjacent G residues. Reducing the number of free G residues decreases their ability to cross-link and form gel structures in the presence of divalent ions [32]. The solution to this problem is to use chemoenzymatic modification of mannuronate [33]. In the first step, the mannuronate is coupled with a polar molecule (e.g. GRGDYP), using carbodiimides. The next step is the production of alginates using epimerases AlgE4 and AlgE6 from *A. vinelandii* or another bacterium [34]. This enables the introduction of G residues (the residues responsible for gelation) into the polymer. Only residues to which the bioactive peptide has not been attached undergo an enzymatic reaction. This approach is one of the methods used to create peptide-modified alginate materials.

The use of alginate materials in vivo requires that, in addition to the necessary biological activity, their antibacterial properties are maintained. Current restrictions on the use of antibiotics, associated with increases in bacterial resistance, have contributed to the increasing interest in materials containing metal compounds with confirmed antibacterial properties [35,36]. Silver is the most often used, but copper compounds are another possibility. Chitosan nanocomposites and chitosan membranes are examples of copper compounds with antibacterial properties [37,38], as are materials based on sodium alginate and copper oxide [39]. Despite the fact that copper is known to have toxic effects when in contact with tissue [40], copper compounds are used in many applications and provide very good antibacterial properties. It has also been shown that the negative electrical charge generated by copper when in contact with the skin reduces the sensation of pain. Copper compounds have been used successfully for the modification of fabrics, achieving very good antibacterial effects [41,42]. As shown by our previous studies [43], it is possible to obtain fibrous structures containing copper embedded in the structure of the material. Copper alginate-based fibers are characterized by high moisture sorption at 35% and retention at 65%. An important property of this type of fibrous structure is also its ability to partially transform into a gel in a humid environment. This enables it to properly protect the area of damaged tissue. Copper alginate hydrogels have very good antibacterial properties, but its direct use is hindered due to their cytotoxicity to mammalian cells [44]. The cytotoxicity of copper alginate hydrogels has been eliminated by the use conjugates of copper alginate with gelatin [45], which indicates that the presence of peptides/proteins in polysaccharide-peptide (protein) materials allows for preserving the properties of alginate with elimination of the adverse effects of Cu^2+^ ions.

The purpose of the current work was to obtain materials derived from copper alginate modified with RGD peptides and to assess their potential for use in the regeneration of damaged skin. It was expected that the materials would have antibacterial activity, resulting from the presence of Cu (II) compounds, but would not be cytotoxic to mammalian cells. The presence of RGD peptides was intended to improve the relevant biological properties of the materials for potential applications in regenerative medicine.

## 2. Materials and Methods 

### 2.1. Formation of Fiber and Nonwovens

**Figure 2:**
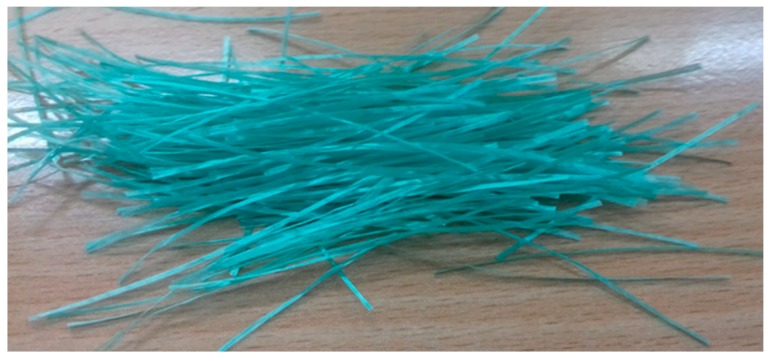
Photograph of the obtained copper alginate fibers.

The process of fiber formation was carried out by the Department of Material and Commodity Sciences and Textile Metrology at Lodz University of Technology. Copper alginate fibers were obtained using the wet method with distilled water as the solvent. A large laboratory spinner with adjustable stretching points was used. A 500-hole spinning nozzle with a hole diameter of 0.08 mm was used in the process. The process of solidifying the copper alginate fibers was carried out in a bath containing a 3% aqueous CuCl_2_ solution, with the addition of a small amount of hydrochloric acid (0.03% HCl) at a temperature of 40 °C. A + 90% filler extract was used. Fiber stretching was performed in two stages. The first stage of the stretching process was completed in a plasticizing bath, identical in composition to the solidifying bath, at a temperature of 67 °C. The second stage of the stretching process was carried out in a superheated steam environment at a temperature of 135–140 °C. After the stretching and rinsing process, the fibers were dried at 25 °C under isometric conditions (Figure 2).

**Figure 3:**
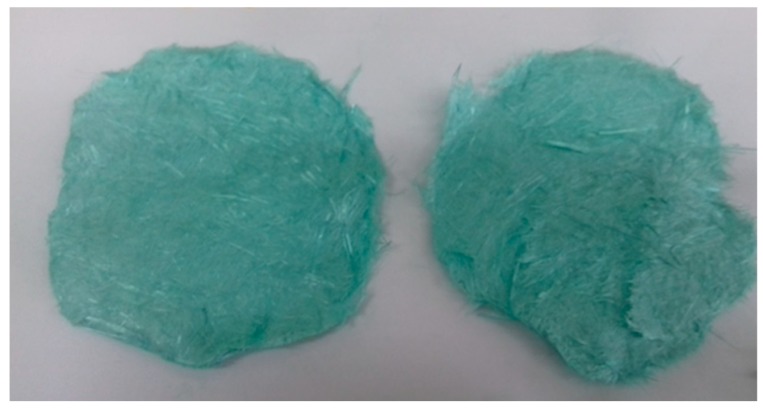
Photograph of nonwovens made of copper alginate fibers.

The fibers were used to obtain a nonwoven fabric, which was made by cutting the fibers, weighing them (0.5 g), and wetting them with distilled water and NaCl (10:1). After thorough mixing, the fibers were transferred to a Büchner funnel. After draining, round nonwoven flakes were obtained with a diameter of 4 cm (Figure 3).

### 2.2. Synthesis of H-RGD-OH and H-RGD-NH_2_

Peptide synthesis was performed under classic solid phase conditions using 2-chlorotrityl resin and Rink-amide resin. Peptides H-RGD-OH and H-RGD-NH_2_ were obtained using Fmoc protective group protocols (see Supporting Materials). Fmoc-protected amino acids were purchased from Novabiochem (San Diego, CA, USA) or Bachem AG (Bubendorf, Switzerland). As a coupling reagent, 4-(4,6-dimethoxy-1,3,5-triazin-2-yl)-4-methylmorpholinium toluene-4-sulfonate (DMT/NMM/TosO^−^) was used [46]. The purity of the isolated crude peptides was >98% (see Supplementary Materials). Analytical reverse-phase high-performance liquid chromatography (RP-HPLC) was performed on an LC Dionex UltiMate 3000 (ThermoFisher Scientific, Waltham, USA), using a Kinetex Reversed Phase C18 column (100 × 4.6 mm) with a flow rate of 0.4 mL/min, eluting with 0.1% trifluoroacetic acid (TFA) in H_2_O (B) and 0.1% TFA in CH_3_CN (A). The HPLC spectra of crude H-RGD-OH and H-RGD-NH_2_ are presented in the Supplementary Materials (Figures S1 and S2). Electron ionization mass spectrometry ESI-MS was carried out on a Bruker microOTOF-QIII (Bruker Corporation, Billerica, MA, USA). Mass spectra of H-RGD-OH and H-RGD-NH_2_ are shown in the Supplementary Materials (Figures S3 and S4).

### 2.3. Preparation of Copper Alginate Nonwovens with Physically Embedded RGD Derivatives

Solutions of H-RGD-OH and H-RGD-NH_2_ peptides at concentrations of 1 mg/mL and 4 mg/mL were used for physical surface modification (spraying method) of the Cu-Alginate. The solutions (10 mL) were prepared by dissolving 10 and/or 40 mg lyophilized H-RGD-OH and/or H-RGD-NH_2_ respectively in Milli-Q water (Merck KGaA, Darmstadt, Germany). Solutions of the dissolved peptides were applied by direct spraying to the copper alginate nonwovens (flakes with a diameter of 4 cm). Complete wetting of the nonwovens was determined by observing the first drops of the solution dripping from the nonwovens placed on the Buchner funnel. The modified nonwovens were dried to a constant weight in a laminar flow chamber at room temperature. In this way, copper alginate nonwovens with physically embedded peptides were obtained: H-RGD-OH (AlgCu-OH 1:1, AlgCu-OH 1:4) and H-RGD-NH_2_ (AlgCu-NH_2_ 1:1, AlgCu-NH_2_ 1:4). The ratios of the copper alginate to RGD derivative were 1:1 and 1:4, respectively.

### 2.4. Preparation of Copper Alginate Nonwovens with Covalently Bound H-RGD-OH (Cu-Alginate-RGD)

H-RGD-OH peptide (86.5 mg, 0.25 mmol), DMT/NMM/TosO^−^ (103.2 mg, 0.25 mmol), and *N*-methyl morpholine (110 µL, 1 mmol) were dissolved in dimethylformamide (DMF) (15 mL). Activation was carried out for 15 min at room temperature. The nonwoven copper alginate (0.4130 g) was treated with the solution of activated RGD. Coupling was carried out for 24 h on a shaker (slow, steady mixing). The stoichiometric ratio of the used substrates was: 1:¼:¼ mmol (copper alginate:peptide:coupling reagent). After 24 h, the nonwoven fabric was washed with DCM (5 mL), DMF (5 mL), DMF-water (5 mL) and CH_3_CN (5 mL), then dried to a constant weight. The same synthetic procedure was used to obtain Ca-Alginate-RGD, which was used in cytotoxicity assessment studies as a comparative material for Cu-Alginate-RGD.

### 2.5. Analysis of Nonwovens Based on Copper Alginate Modified with RGD Peptides

FTIR ATR Spectroscopy: Infra-red spectra were obtained using a VERTEX 70 FTIR Spectrometer (Bruker, Bremen, Germany) with an ATR Golden Gate Diamond Accessory (Specac, UK). Absorption spectra at a resolution of one data point per 2 cm^−1^ were obtained in the region between 400 cm^−1^ and 4000 cm^−1^. A total of 64 scans were collected, Fourier-transformed and averaged for each measurement. Recording of the spectra was performed using Bruker OPUS software (Version 6.5).

Raman spectroscopy: Raman spectroscopy was carried out in the closed microscope chamber of the spectroscope, with the samples placed on a microscope plate. The samples were positioned in the laser light focus by means of a microscope (magnification 50×) with a charge-coupled device (CCD) camera. Raman spectra were obtained using a Renishaw InVia Reflex dispersive spectrometer with a Leica microscope (Renishaw, Wotton-under-Edge, UK). An excitation source of λ = 785 nm, 300 mW was applied, with a spectral resolution of 1 cm^−1^. The spectra were accumulated over a 10 s integration time. Laser power was dependent on the sample and varied from 1% to 10% of power. The spectra were recorded using Renishaw WIRE 3.2 software (Version 3.2).

Scanning Electron Microscopy/Energy Dispersive Spectroscopy (SEM/EDS) analysis: Microscopic measurements of the surface topography of the materials were carried out on a VEGA3 TESCAN (TESCAN ORSAY HOLDING, Brno, Czech Republic) scanning electron microscope equipped with an electron optics system, enabling observation of the surfaces of the materials at a 4–1,000,000-times magnification across an energy range of the incident electron beam from 0.2 keV to 30 keV with maximum resolutions of 3 nm in secondary electron emission mode and 3.5 nm in backscattered electron mode. Scanning electron microscope (SEM) images of the surface topography and cross-sections were taken using a high vacuum mode, 20 keV probe energy. The surface of each preparation was sprayed with a conductive substance—gold—using a vacuum sputter (Quorum Technologies Ltd., Lewes, UK). For surface topography studies, magnifications of 300×, 1000×, 5000×, 10000× and 20,000× were used. Elemental analysis was performed using EDS INCA Energy X-ray energy dispersion spectrometer from Oxford Instruments (Abingdon, UK).

### 2.6. Testing the Antibacterial Properties of Materials Based on Cu-Alginate-RGD Conjugates

Two types of materials were used in this study: Ca-Alginate-RGD (control, covalent bound RGD peptide with calcium alginate) and Cu-Alginate-RGD (covalent bound RGD peptide with copper alginate). Antibacterial activity tests were performed according to the ISO 20743:2013 standard. Six control samples and six samples with an antibacterial compound were used. The test samples were placed in separate sterile containers and treated with 70% ethanol for a period of 30 min prior to the test. The further sterilization was performed by irradiation of both sides of the sample with UV-C light for 30 min. In accordance with the requirements of ISO 20743: 2013, the following bacterial strains were used:

- *Staphylococcus aureus* American Type Culture Collection (ATCC) 6538,
- *Klebsiella pneumoniae* ATCC 4352.

Suspensions with densities from 1.0 × 10^5^ to 3.0 × 10^5^ CFU/mL were prepared for each of the strains. Separate studies were conducted for each material. Test samples were inoculated with a bacterial suspension using 0.2 mL of the suspension for each sample. The suspension was applied to the sample in several places using a pipette. Immediately after inoculation, 20 mL of neutralizer was added to each of six samples of the control material and six samples of Cu-Alginate-RGD. Six samples (three control samples of Ca-Alginate-RGD and three test samples of Cu-Alginate-RGD) were seeded on tryptone soya agar (TSA) medium at dilutions of 100 to 10^−4^ to determine the initial number of bacteria per sample. Another six samples (three control samples of Ca-Alginate and three test samples Cu-Alginate-RGD) were incubated at 37 ± 2 °C for 24 h. After incubation, 20 mL of neutralizer was again added to the samples. The samples were seeded on TSA medium at dilutions of 100 to 10^−4^ to determine the final count bacteria per sample.

### 2.7. Cytotoxicity Studies

Cell culture materials were sterilized by immersion in 70% ethanol for a period of 30 min. Subsequent sterilization consisted of irradiating both sides of the sample with UV-C light for a period of 30 min. Just before loading into the culture well, the material was washed with sterile phosphate-buffered saline (PBS, HyClone, Pittsburgh, PA, USA). The mouse fibroblast cell line L929 (ATCC, Manassas, VA, USA) was grown in 75 cm^2^ bottles (Nunc™, Roskilde, Denmark) using Eagle’s Minimum Essential Medium (EMEM, ATCC, Manassas, VA, USA), supplemented with 10% horse serum (ATCC, Manassas, VA, USA). Cell culture conditions: 37 °C, atmosphere enriched with 5% CO_2_. The medium was changed every 3 days until a monolayer was formed. Cells for further testing were transferred using 5% trypsin-EDTA (HyClone, Pittsburgh, PA, USA). The prepared materials were placed at the bottom of the culture wells (Nunc™, Roskilde, Denmark). The L929 cells were seeded on well films with 1.5 10^4^ cells/mL/well and cultured for 1, 3, and 7 days. Tissue culture polystyrene (TCPS) plates served as a positive control.

Determination of viability—Presto Blue test

Supernatant was pipetted from the wells containing cells cultured on samples of material as well as from the control wells, returning 270 µL of supernatant to each well. Next, 30 µL of PrestoBlue reagent was added to each well. The wells were then incubated for 120 min in a culture incubator.

After incubation, 2 × 100 µL of supernatant from each of the samples of material, and the control was transferred to each of the 96 wells of an optical plate (Optiplate, Perkin-Elmer, Waltham, MA, USA). The fluorescence intensity was measured using a microplate reader. Fluorescence intensity was calculated on the sample surface (culture well) with the test material. Statistical analysis was performed using one-way analysis of variance (ANOVA). Results with *p* > 0.05 were considered to be statistically significant.

## 3. Results and Discussion

### 3.1. Preparation of Fibers and Nonwovens Based on Copper Alginate 

**Figure 4:**
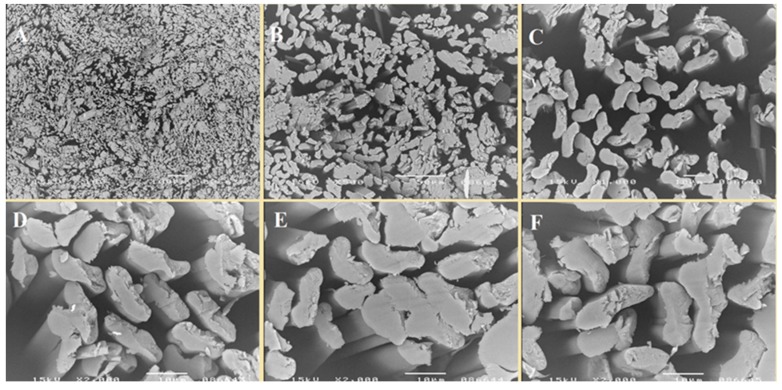
SEM images. Transverse view of copper alginate fibers at magnifications: (**A**) 100×, (**B**) 500×, (**C**) 1000×, (**D**–**F**) 2000×.

**Figure 5:**
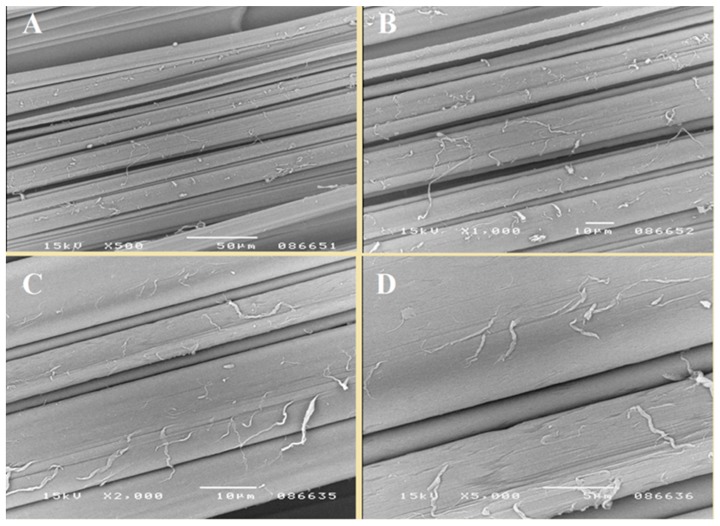
SEM images. Longitudinal view of copper alginate fibers, enlargement: (**A**) 500×, (**B**) 1000×, (**C**) 2000×, (**D**) 5000×.

**Figure 6:**
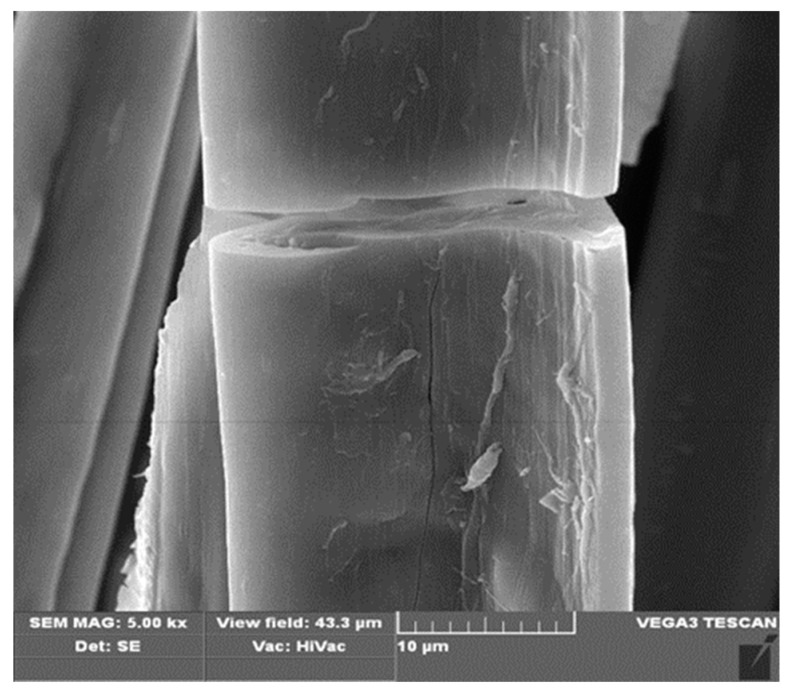

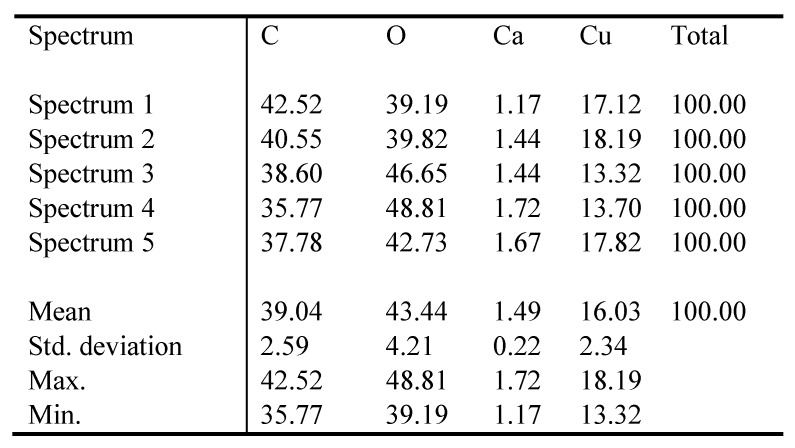
SEM microscope images with EDS analysis of copper alginate fibers.

In the first stage of the research, copper alginate fibers were obtained by the wet solution method (in water) using a large laboratory spinner and a 3% solution of CuCl_2_. The structures of the fibers were subjected to microscopic examination using a SEM microscope (Figure 4, Figure 5 and Figure 6).

The fibers were characterized by the numerous cracks, scratches, and crevices, which are typical for fibers formed by the wet solution method. However, this irregular surface is attractive from the point of view of surface modification with short peptides. At the same time, EDS analysis (the average of 5 samples) revealed 16.03% (Std. Deviation = 2.34) of the copper (Figure 6).

The copper alginate fibers were used to obtain nonwovens in the form of flakes with a diameter of 4 cm. Nonwovens were used as a substrate for the physical and chemical attachment of RGD derivatives. Comparison of two methods for attaching a biologically active peptide to a polysaccharide matrix should enable assessment of whether the functionalization method affects the biological activity of conjugates. Moreover, it may be expected that different functionalization methods could influence the stability of bonded peptides. In the case of a conjugate with physically embedded peptides, binding is caused only by weak interactions (hydrogen, ionic, and other bonds), while chemical functionalization should lead to significantly more stable connections, since the peptide is attached to the polysaccharide by a permanent covalent bond. Two RGD derivatives with a free carboxyl group (H-RGD-OH) and in the form of an amide (H-RGD-NH_2_) were used in order to investigate whether, in the case of polysaccharide materials modified with RGD peptides, derivatives with an amide function at the C-terminus would be more active compared to H-RGD-OH [47].

### 3.2. Functionalization of Copper Alginate Using RGD Derivatives

The RGD derivatives used for physical modification of the copper alginate surfaces have a C-terminal amino acid in the form of an acid or amide: H-RGD-OH and H-RGD-NH_2_ peptides. The peptides were applied to the surfaces of the nonwoven materials using the spraying method. Two different concentrations of the peptides (1 mg/mL and 4 mg/mL) were applied. This enabled the preparation of copper alginate conjugates with physically embedded H-RGD-OH peptides (AlgCu-OH 1:1, AlgCu-OH 1:4) and H-RGD-NH_2_ (AlgCu-NH_2_ 1:1, AlgCu-NH_2_ 1:4) with ratios copper alginate to RGD derivative of 1:1 and 1:4.

**Figure 7:**
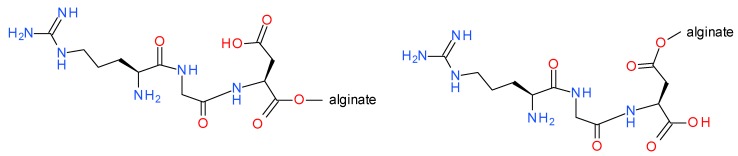
Examples of isomeric RGD conjugates with alginic acid salts.

Attempts were also made to obtain copper alginate modified with the H-RGD-OH derivative, which is covalently linked to the polysaccharide matrix. The H-RGD-OH peptide was attached to the polysaccharide using 4-(4,6-dimethoxy-1,3,5-triazin-2-yl)-4-methylmorpholinium toluene-4-sulfonate (DMT/NMM/TosO^−^) [44] as a coupling reagent. This reagent has been used previously for the successful synthesis of peptides as well as esters [46,48], and also for the synthesis of conjugates of polysaccharide with peptides [49,50,51,52,53,54]. In addition to the high efficiency of this reagent, it has the very important advantage of forming 2-hydroxy-4,6-dimethoxy-1,3,5-triazine as a by-product, which can be easily removed by extraction with both organic solvents and aqueous solutions. This prevents formation of insoluble deposits on the surfaces of the modified materials. Given the structural variability of alginates (MG, GG, MM blocks) and the presence of two carboxyl groups in the H-RGD-OH peptide, it is possible to obtain a variety of isomeric products (Figure 7).

The preferential formation of ester bonds between the hydroxyl groups of copper alginate and the carboxyl groups of H-RGD-OH results from the fact that synthesis proceeds under anhydrous conditions, which prevent the displacement of Cu^2+^ cations by monovalent ions (no release of copper salts was observed). Moreover, the peptide H-RGD-OH was activated with DMT/NMM/TosO^−^, which resulted in the formation of superactive triazine esters. Thin-layer chromatography and 4-(4-nitrobenzyl)pyridine (NBP) [55] were used to visualize the triazine esters being formed and the simultaneous disappearance of DMT/NMM/TosO^−^. Two methods were used to assess the efficiency of conjugation of the copper alginate with the RGD derivatives: FTIR and Raman spectroscopy.

**Figure 8:**
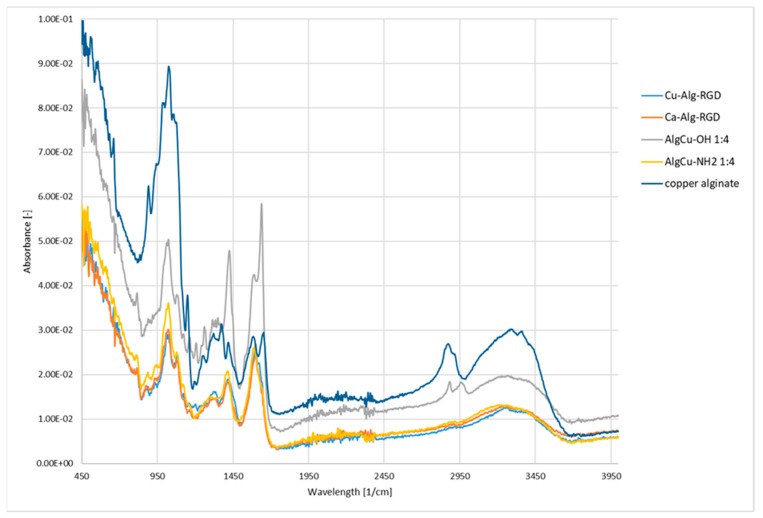
FTIR spectra of copper alginate conjugates with RGD derivatives.

The FTIR studies began with an analysis of unmodified copper alginate. A characteristic wide band was found in the vibration range of 3500–3100 cm^−1^, which is typical for stretching vibrations of OH groups blocks M and G (Figure 8). In addition, two absorption bands were observed which correspond to the valence vibrations of the C-O group present in the carboxylate ion (asymmetrical at 1420 cm^−1^ and symmetrical at 1620 cm^−1^). The band in the 1050 cm^−1^ range with the highest intensity corresponds to the skeletal vibrations of the M and G pyran rings and is characteristic of the polysaccharide glycosidic moiety.

Analysis of the spectra of the copper alginate conjugates modified with RGD peptides (both physically and chemically) confirms the presence of 1650 cm^−1^ and 1550 cm^−1^ bands characteristic for peptide bonds (Figure 8). Additional confirmation of the successful modification of the copper alginate with peptides is also provided by the presence of a band at 1300 cm^−1^ characteristic for peptide (protein) compounds. Unfortunately, other characteristic bands for peptides/proteins in the 3500–3200 cm^−1^ range are poorly visible, due to the presence of intense bands typical for saccharides in this range (typical stretching vibrations of OH groups).

**Figure 9:**
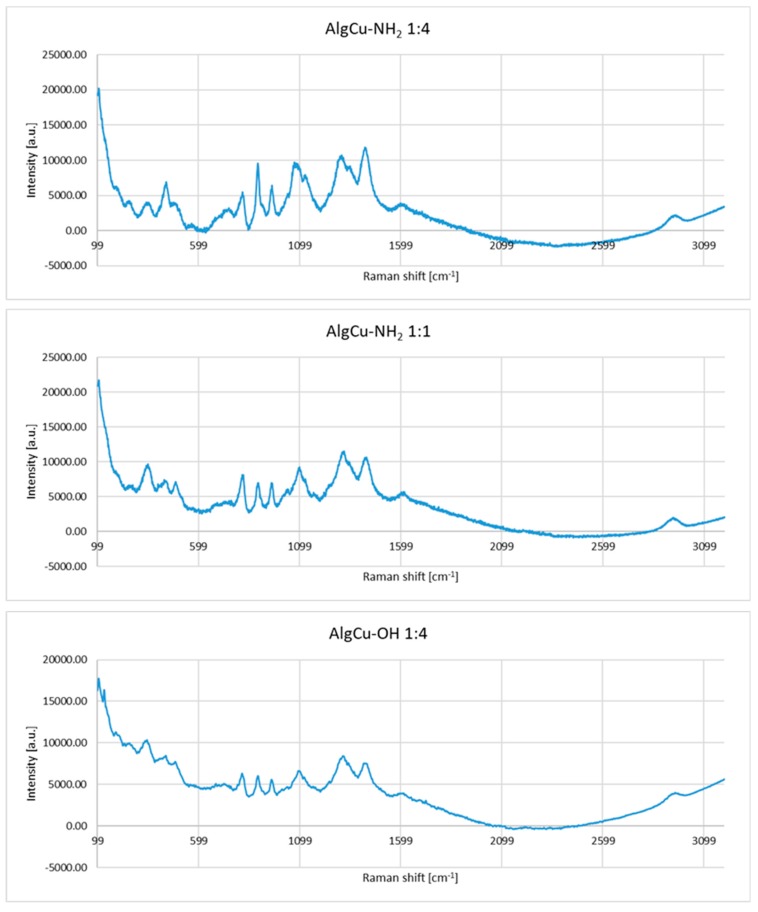

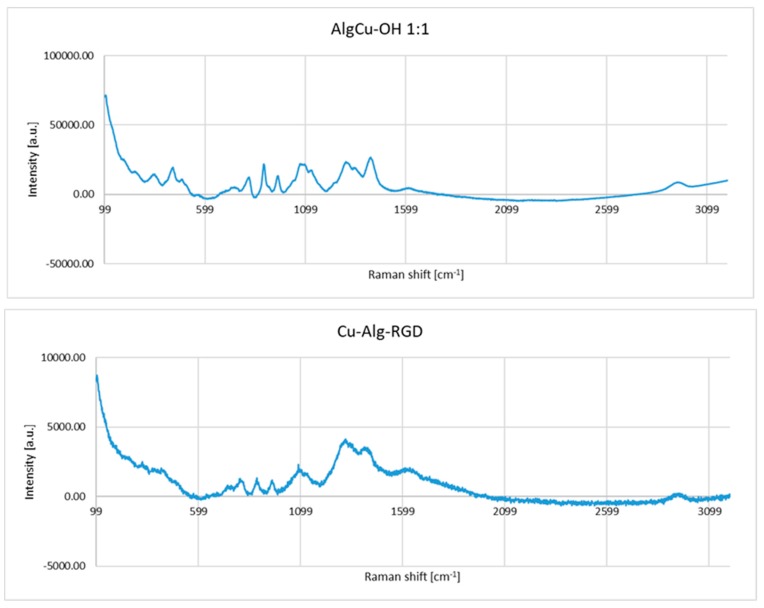
Raman spectra of copper alginate conjugates with RGD derivatives.

In the Raman spectra of the copper alginate conjugates with RGD derivatives (AlgCu-NH_2_ and AlgCu-OH) obtained by physical modification, changes in the course and intensity of the bands are visible in the range of 1180–1020 cm^−1^. This is the range of typical polysaccharide vibrations (C-O-C glycoside bond and sugar ring) (Figure 9), which indicates a different molar ratio of peptides applied to the polysaccharide nonwoven fabric.

The second characteristic area of the spectrum that confirms the effectiveness of modification is in the region of 1583–1484 cm^−1^, which is characteristic for the amide bond (amide II) derived from RGD derivatives. In the case of the Cu-Alg-RGD conjugate which resulted from chemical modification, the range of changes is similar and relates to vibrations associated with the sugar substrate and the presence of amide bands. The intensity of the bands in the regions 1718–1584 cm^−1^ (amide I) and 1484–1583 cm^−1^ (amide II) confirms the chemical binding of RGD peptide to the alginate substrate (Figure 9).

### 3.3. Evaluation of Antibacterial Activity

The purpose of this study was to determine the antibacterial activity of Cu-Alg-RGD material (covalently attached RGD peptide to copper alginate) compared to that of calcium alginate covalently modified with RGD (Ca-Alg-RGD), which was used as a reference. According to the criteria specified in the ISO 20743: 2013 standard, a material has significant antibacterial activity when the value for antibacterial activity A is in the range 2 ≤ A <3. When A > 3, the material is classified as highly antibacterial. The following required ISO 20743: 2013 standards were met in the tests performed using Cu-Alg-RGD as anti-bacterial material: (a) the inoculum concentration ranged from 1 × 10^5^ to 3 × 10^5^; (b) the maximum difference for the three controls tested immediately after inoculation was ≤1; (c) for the control sample, the value of the increase F was ≥1. Compliance with the required conditions allowed determination of the A value for Cu-Alg-RGD (Table 1).

The results of Cu-Alginate-RGD antibacterial activity were assessed at A = 2.90 for *S. aureus*, which indicates significant antibacterial activity. For *K. pneumoniae*, A = 4.56, which indicates strong antibacterial activity of the product.

### 3.4. Cytotoxicity Studies 

**Figure 10:**
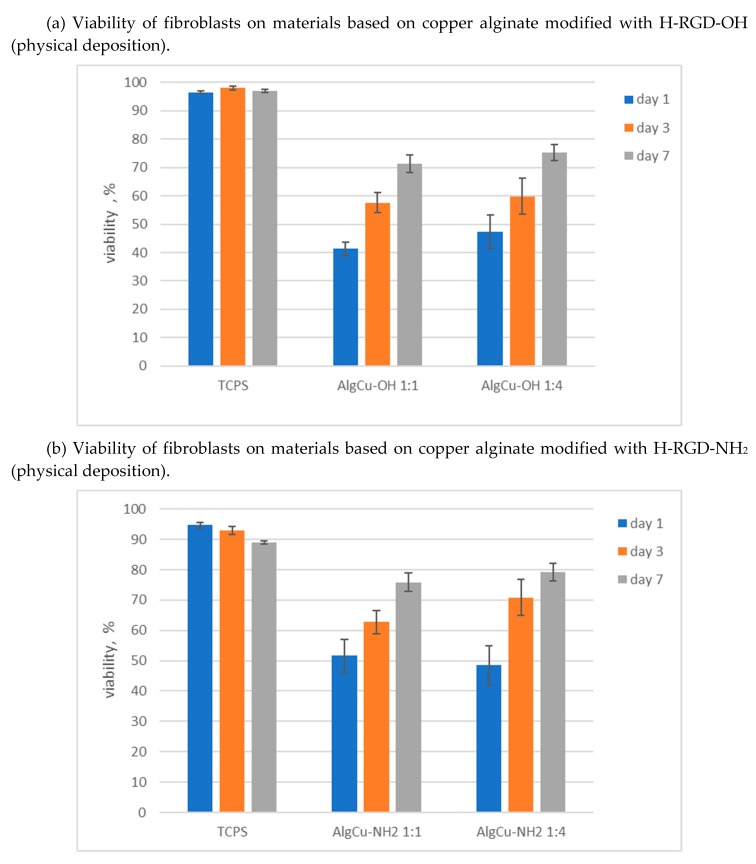

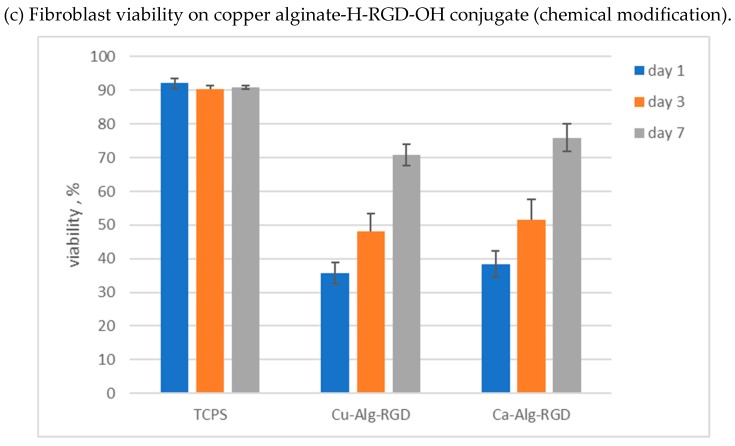
Viability of L929 mouse fibroblast cells in the presence of copper alginate conjugates with RGD derivatives.

The L929 mouse fibroblast cell line was used in cytotoxicity assessments. Studies were conducted over seven days, with viability assessed after 1, 3 and 7 days. Presto Blue test was used, allowing assessment of the viability and proliferation of a wide range of cell types [56]. PrestoBlue™ reagent is quickly reduced by metabolically active cells, providing a quantitative measure of viability and cytotoxicity. The results of studies on the survival of fibroblasts growing on materials based on copper alginate modified with RGD derivatives are shown in Figure 10. The cytotoxicity assays were performed in five replications, and the results presented are average values. The low standard deviation confirms the high reproducibility of the test. Statistical analysis was performed using one-way analysis of variance (ANOVA). Results with *p* > 0.05 were considered to be statistically significant.

It was found that copper alginate conjugates with RGD derivatives are not cytotoxic to the L929 mouse fibroblast cell line. This observation applies to all the tested materials, both the derivatives obtained as a result of the physical deposition of H-RGD-OH and H-RGD-NH_2_ and the material with a chemically bound RGD peptide. In the case of materials obtained as a result of physical deposition of RGD derivatives, the amount of the peptide component had no significant effect on cell survival (Figure 10a,b). Only an insignificant effect of the amount of deposited RGD derivative is seen in the case of H-RGD-OH on the first day of measurement (Figure 10a), where a higher peptide concentration increases the survival rate by 6%. This effect is not visible in the case of H-RGD-NH_2_ (Figure 10b), which may indicate a higher activity of a derivative with a blocked carboxyl group of the C-terminal amino acid (this observation is consistent with the literature data [57]). It is interesting to note that, in all cases, significantly lower cell survival was observed in the initial incubation phase than in the final phase. Thus, it can be postulated either that copper compounds have a greater adverse (cytotoxic) effect in the initial incubation phase, or that this effect may be caused by the alginate, as a similar result was observed for Ca-Alg-RGD (Figure 10c). The decisive effect of RGD on the elimination of the harmful influence of Cu^2+^ ions may be evidenced by the fact that our results are inverse to those described by Wimonwan and Pitt [44], where an increase of the cytotoxicity of copper alginate was observed during the incubation process. An additional fact confirming the effectiveness of RGD is that derivatives of both calcium alginate and copper alginate with covalently attached RGD peptide are more cytotoxic in the initial phase compared to materials with physically embedded RGD, which may indicate that it is necessary to release biologically active peptides from the polysaccharide matrix to get a positive effect of RGD. For covalently linked Cu-Alg-RGD and Ca-Alg-RGD derivatives, prior ester bond hydrolysis is necessary. More research is needed to explain the mechanism by which the cytotoxic effect on cells may be reduced, as, in terms of their high antibacterial activity, the final materials seem very interesting from the point of view of regenerative medicine.

## 4. Conclusions

In this study, copper alginate fibers were formed using the standard wet method, with distilled water as a solvent. The fibers contained 16.03% copper based on constant weight. The alginate fibers were used to obtain copper alginate nonwovens. The nonwovens were used as solid matrices into which were introduced RGD derivatives. Arginine-glycine-aspartate derivatives can positively affect the entire spectrum of processes in cells, from the stimulation of cell adhesion to cell migration, proliferation, survival, and apoptosis. Copper alginate conjugates with RGD derivatives were obtained by deep spraying and covalent attachment of the peptide to the polysaccharide matrix. Two types of material were obtained: (a) nonwovens with RGD derivatives H-RGD-OH and H-RGD-NH_2_ physically embedded into the matrix, and (b) nonwovens with the RGD derivative covalently bound to the polysaccharide matrix via an ester bond. Triazine coupling reagent was used to covalently attach the H-RGD-OH to the copper alginate. The effectiveness of both modification procedures was confirmed by spectroscopic methods (FTIR and Raman).

Studies using *Staphylococcus aureus* ATCC 6538 and *Klebsiella pneumonie* ATCC4352 showed that Cu-Alginate-RGD (conjugate with a chemically bonded peptide) has antibacterial activity, assessed at A = 2.90 for *S. aureus*, which indicates significant antibacterial activity. For *K. pneumoniae*, A = 4.56, which indicates strong antibacterial activity.

Cytotoxicity studies on the L929 mouse fibroblast cell line using the Presto Blue test showed that the copper alginate conjugates with RGD derivatives are not cytotoxic. During incubation, an increase in cell survival was observed, which indicates that there were no adverse effects due to the presence of copper ions. Given that they also meet the requirements of biocompatibility and antibacterial activity (there is no need to introduce additional compounds to provide antimicrobial activity), materials based on copper alginate conjugates with biologically active peptides show great potential for use in regenerative medicine.

## Figures and Tables

**Table 1:** Comparison of the results of antibacterial activity tests for Cu-Alg-RGD.

The Value of Antibacterial Activity for the Seeding Method		
Bacteria	*Staphylococcus aureus* ATCC 6538	*Klebsiella pneumonie* ATCC 4352
Inoculum concentration (colony-forming unit (CFU)/mL)	2.6 × 10^5^	1.0 × 10^5^
Value of growth F (F = log C_T_−log C_0_)	1.75	2.75
Value of growth G (G = log T_T_−log T_0_)	−1.15	−1.81
Value of antibacterial activity A (A = F−G)	2.90	4.56

C_T_—number of bacteria in the control sample after incubation, C_0_—number of bacteria in the control sample before incubation, T_T_—number of bacteria in Cu-Alg-RGD material after incubation, T_0_—number of bacteria in Cu-Alg-RGD before incubation.

## Supplementary Materials

The following are available online at https://www.mdpi.com/1996-1944/13/2/337/s1, Figure S1: HPLC of H-Arg-Gly-Asp-OH, Figure S2: HPLC of H-Arg-Gly-Asp-NH_2_, Figure S3: MS spectrum of H-Arg-Gly-Asp-OH, Figure S4: HPLC of H-Arg-Gly-Asp-NH_2_, description of the synthesis of RGD derivatives.
