# Supplementary material for: Conjugates of Copper Alginate with Arginine-Glycine-Aspartic Acid (RGD) for Potential Use in Regenerative Medicine
Source: Materials (Basel). 2020 Jan 11;13(2):337. doi: 10.3390/ma13020337 (PMC7013949; doi:10.3390/ma13020337)
Supplement: Supplementary file 1 [file materials-13-00337-s001.pdf]

materials

# Conjugates of Copper Alginate with Arginine-Glycine-Aspartic Acid (RGD) for Potential use in Regenerative Medicine

**Justyna Fraczyk [1], Joanna Wasko [1], Malgorzata Walczak [1], Zbigniew J. Kaminski [1], Dorota Puchowicz [2], Irena Kaminska [2], Maciej Boguń [2], Marcin Kolasa [3], Ewa Stodolak-Zych [4] and Anna Scislowska-Czarnecka [5] and Beata Kolesinska [1,*]**

1. Institute of Organic Chemistry, Faculty of Chemistry, Lodz University of Technology, Zeromskiego 116, 90-924 Lodz, Poland; justyna.fraczyk@p.lodz.pl (J.F.); joanna.wasko@edu.p.lodz.pl (J.W.); malgorzata.walczak@p.lodz.pl (M.W); zbigniew.kaminski@p.lodz.pl (Z.J.K.)
2. Lukasiewicz Research Network-Textile Research Institute, Brzezinska 5/15, 92-103 Lodz, Poland; puchowicz@iw.lodz.pl (D.P.); ikaminska@iw.lodz.pl (I.K.); mbogun@iw.lodz.pl (M.B)
3. Military Institute of Hygiene and Epidemiology Department of Pharmacology and Toxicology, Kozielska 4, 01-163, Warsaw, Poland; marcinkolasa@wp.pl
4. AGH-University of Science and Technology, Department of Biomaterials, A. Mickiewicz 30, 30-059 Krakow, Poland; stodolak@agh.edu.pl (E.S.Z.)
5. Academy of Physical Education, Department of Physiotherapy, Section of Anatomy, Krakow, Poland; anna.scislowska@awf.krakow.pl (A.S.C.)

* Correspondence: beata.kolesinska@p.lodz.pl; Tel.: +48-42-631--3162 (B.K.)

## *General Methods for Solid-Phase Peptide Synthesis*

### *Method 1a. Attachment of C-Terminal Amino Acid to the 2-Chlorotrityl Chloride Resin*

At room temperature, 1 g of 2-chlorotrityl chloride resin (CTC) with maximum loading of 1 mmol/g was preswollen for 30 min in dry dichloromethane (DCM). Subsequently, 3 eq. of the appropriate Fmoc-Asp(tBu)-OH was dissolved in DCM, to which 6 eq. *N*,*N*-diisopropylethylamine (DIPEA) was added. This loading solution was added to 2-chlorotrityl chloride resin and the suspension was shaken for 2 h. After this time, three washings steps with DCM were performed. To cap unreacted active groups on the 2-chlorotrityl chloride resin, a solution of DCM:MeOH:DIPEA (17:2:1) was prepared and added to the previously washed and drained resin. This suspension was shaken for 30 min at room temperature. The capping solution was then filtered, and the resin was washed three times with *N*,*N*-dimethylformamide (DMF) and three times with DCM.

### *Method 1b. Attachment of C-Terminal Amino Acid to the Rink Amide Resin*

The resin (0.5 g) was swollen in DMF and a solution of 20% piperidine in DMF is added to cleave the resin-bound Fmoc groups. The reaction mixture is shaken for 20 min at room temperature. The resin is drained and washed with DMF (5x). A solution of 3.0 equiv of amino acid (relative to resin capacity) in DMF is added to the resin. The reaction mixture is shaken at room temperature for 5 min. A solution of 3.0 equiv DMT/NMM/$TosO^-$ in DMF was added to the reaction mixture, followed by 6.0 equiv of DIPEA. The reaction mixture is shaken for 45 min at room temperature. The resin was drained and washed with DMF (3x), DCM (3x), MeOH (3x). Small quantities of resin are tested for unreacted amine with Kaiser test.

### Standard Coupling Procedure (Method 2)

DMF solution of 3 equiv. of amino acid, 3 equiv. of coupling reagent (DMT/NMM/$TosO^-$) and 6 equiv. of NMM was added to the resin and shaken for 1-2h or overnight. The progress of the reaction was monitored by the Kaiser test.

### Deprotection of Fmoc group (Method 3)

The Fmoc group was removed by treating of 25% piperidine in DMF (15-20 min).

Kaiser test (Method 4)

Solution 1: 5 g ninhydrin in 100 mL EtOH. Solution 2: 80 g PhOH and 20 mL EtOH. Solution 3: 2 mg of KCN in 100 mL pyridine. To a resin beads 2-3 drops of each solution was added. The suspension was heat for 2-3 min at 100°C.

Cleavage from the Resin (Method 5)

Peptides were cleaved from the resin using TFA/TIS/H2O (95:2.5:2.5; ca. 2ml/0.1g resin). Cleavage was achieved during 4 h. After precipitation and liophilization The final product was obtained as TFA salt.

Synthesis of H-Arg-Gly-Asp-OH

Fmoc-Asp(OtBu)-OH (0.617 g, 1.5 mmol) was attached to 2-chlorotrityl chloride resin (500 mg, 1.0 mmol/g) according to Method 1a. Starting materials: 2-chlorotrityl chloride resin (0.5 g, 1.0 mmol/g, 0.5 mmol), Fmoc-Asp(OtBu)-OH (0.617 g, 1.5 mmol), DIPEA (540 μL, 3.0 mmol), Fmoc-Gly-OH (0.446 g, 1.5 mmol), Fmoc-Arg(Pbf)-OH (0.973 g, 1.5 mmol), DMT/NMM/TosO$^-$ (0.619 g, 1.5 mmol) and NMM (330 μL, 3.0 mmol). The peptide was cleaved from the resin according to Method 5. Product: H-Agr-Gly-Asp-OH-OH. Anal. RP-HPLC (20–60% B in 45 min): $t_R$ 2.15 min, purity 99.4%. LC/MS: 347.1807 corresponding to $[M+H]^+$ ($M^+$ for $C_{12}H_{22}N_6O_6^+$; calc. 346.3397).

Synthesis of H-Arg-Gly-Asp-NH2

Fmoc-Asp(OtBu)-OH (0.617 g, 1.5 mmol) was attached to Rink Amide resin (500 mg, 1.0 mmol/g) according to Method 1b. Starting materials: Rink Amide resin (0.5 g, 1.0 mmol/g, 0.5 mmol), Fmoc-Asp(OtBu)-OH (0.617 g, 1.5 mmol), DIPEA (540 μL, 3.0 mmol), Fmoc-Gly-OH (0.446 g, 1.5 mmol), Fmoc-Arg(Pbf)-OH (0.973 g, 1.5 mmol), DMT/NMM/TosO$^-$ (0.619 g, 1.5 mmol) and NMM (330 μL, 3.0 mmol). The peptide was cleaved from the resin according to Method 5. Product: H-Agr-Gly-Asp-NH2. Anal. RP-HPLC (20–60% B in 45 min): $t_R$ 2.09 min, purity 99.7%. LC/MS: 346.1963 corresponding to $[M+H]^+$ ($M^+$ for $C_{12}H_{23}N_7O_5^+$; calc. 345.1601).

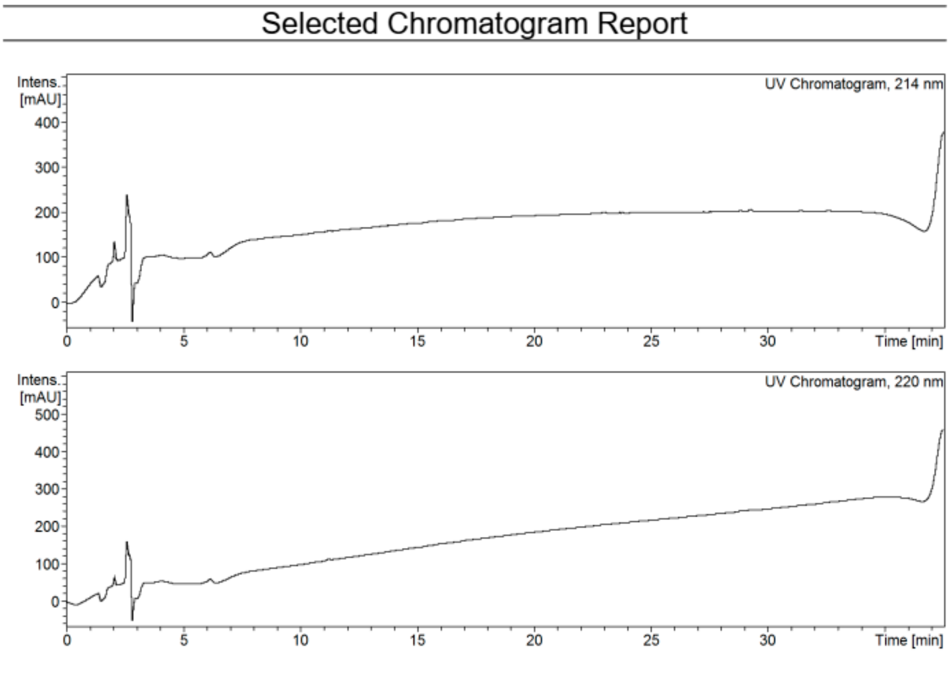


**Figure S1.** HPLC of H-Arg-Gly-Asp-OH.

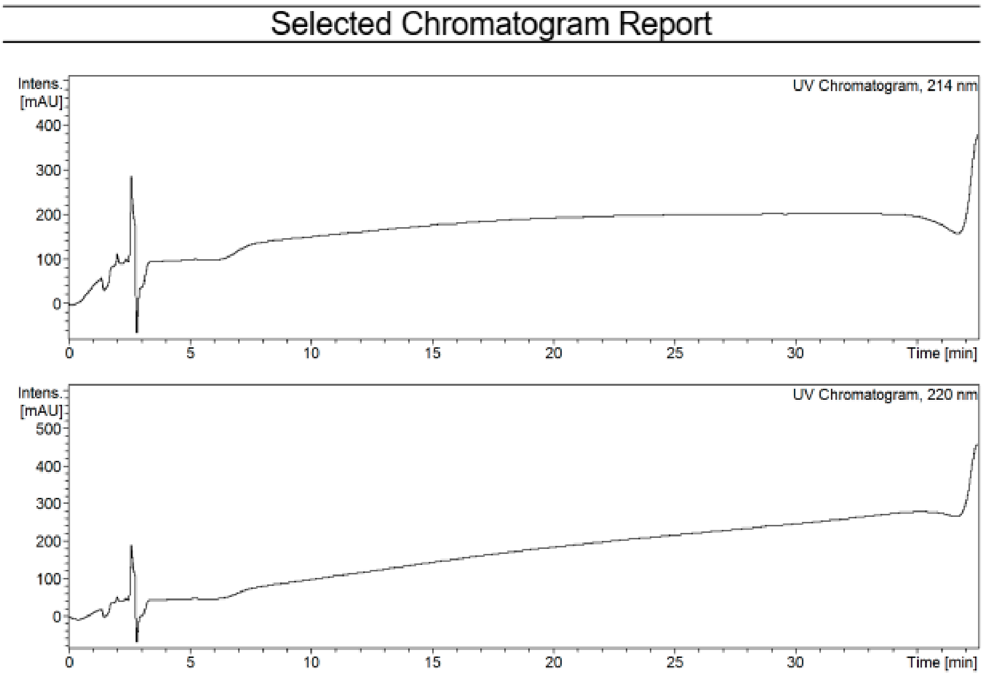


**Figure S2.** HPLC of H-Arg-Gly-Asp-$NH_2$.

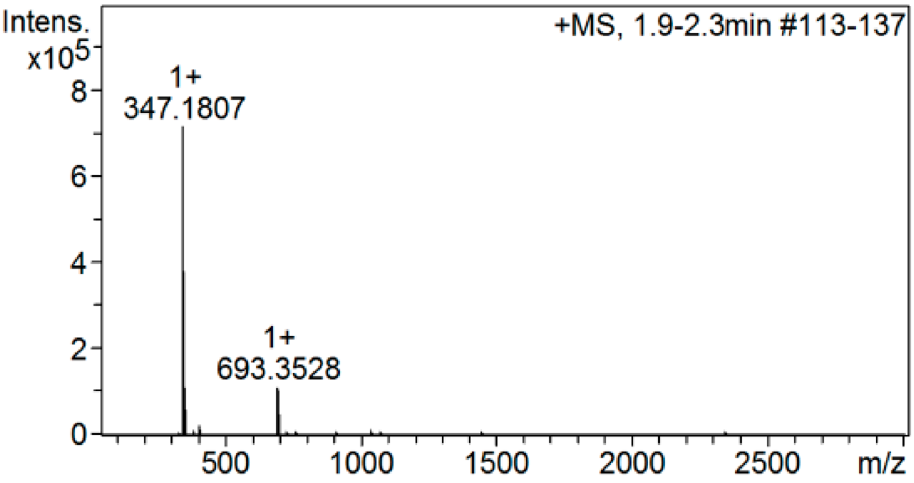


| # | m/z | I |
|---|---|---|
| 1 | 347.1807 | 715184 |
| 2 | 348.1817 | 112393 |
| 3 | 349.1823 | 16481 |
| 4 | 385.1234 | 8190 |
| 5 | 387.2104 | 5989 |
| 6 | 404.1995 | 21989 |
| 7 | 693.3528 | 112918 |
| 8 | 694.3551 | 30930 |
| 9 | 763.0614 | 5807 |
| 10 | 1039.5228 | 9165 |

**Figure S3.** MS spectrum of H-Arg-Gly-Asp-OH.

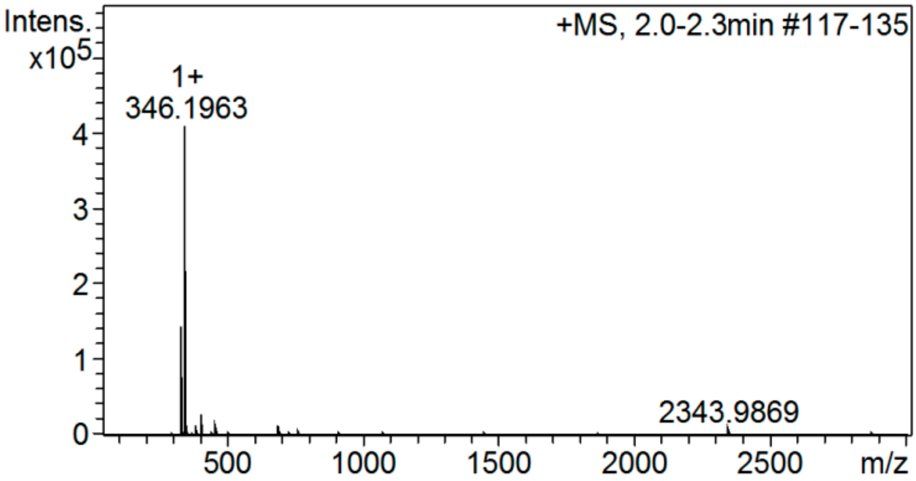


| # | m/z | I |
|---|---|---|
| 1 | 329.1680 | 143408 |
| 2 | 330.1681 | 18830 |
| 3 | 346.1963 | 408751 |
| 4 | 347.1956 | 61597 |
| 5 | 348.1954 | 8046 |
| 6 | 384.1427 | 13983 |
| 7 | 386.2084 | 13645 |
| 8 | 403.2193 | 27552 |
| 9 | 460.2431 | 11440 |
| 10 | 691.3811 | 11704 |

**Figure S4.** MS spectrum of H-Arg-Gly-Asp-$NH_2$.

© 2020 by the authors. Submitted for possible open access publication under the terms and conditions of the Creative Commons Attribution (CC BY) license (http://creativecommons.org/licenses/by/4.0/).
